# Integration of Next-Generation Sequencing in Diagnosing and Minimal Residual Disease Detection in Patients With Philadelphia Chromosome-Like Acute Lymphoblastic Leukemia

**DOI:** 10.7759/cureus.10696

**Published:** 2020-09-28

**Authors:** Nazleen Sherali, Tariq Hamadneh, Saba Aftab, Michael Alfonso, Nicholas Tsouklidis

**Affiliations:** 1 Medicine, Liaquat University of Medical and Health Sciences, Jamshoro, PAK; 2 Medicine, California Institute of Behavioral Neurosciences & Psychology, Fairfield, USA; 3 Ophthalmology, The Third Affiliated Hospital of Southern Medical University, Guangzhou, CHN; 4 Ophthalmology, California Institute of Behavioral Neurosciences & Psychology, Fairfield, USA; 5 Medicine, Hamdard College of Medicine and Dentistry, Karachi, PAK; 6 Health Care Administration, University of Cincinnati Health, Cincinnati, USA; 7 Medicine, Atlantic University School of Medicine, Gros Islet, LCA

**Keywords:** next-generation sequencing, philadelphia chromosome like acute lymphoblastic leukemia, acute lymphoblastic leukemia, bcr-abl like all, minimal residual disease

## Abstract

Philadelphia-like (Ph-like) acute lymphoblastic leukemia (ALL) is a high-risk subtype of B cell ALL. It accounts for 20% of all B cell ALL cases and is similar to BCR-ABL1 in gene expression profile but lacks BCR-ABL fusion. It is highly heterogeneous and is characterized by genetic alterations that activate kinase and cytokine receptor signaling. Most of these alterations are amenable to tyrosine kinase inhibitors. Ph-like ALL is prevalent in pediatric and young adults, more common in males, and frequently seen in patients with Hispanic ancestry. It is associated with inadequate response to induction therapy, high minimal residual disease (MRD) levels, and increased risk of relapse. Overall survival and event-free survival are also inferior in these patients as compared to non-Ph-like ALL. In the clinical practice, low-density array, real-time quantitative polymerase chain reaction (RQ-PCR), flow cytometry, fluorescence in situ hybridization are used to identify genetic alteration in these patients. With the advent of next-generation sequencing (NGS), our understanding of disease pathogenesis and precision medicine has been improved. In this review, we analyzed data from several studies that used NGS as one of the diagnostic methods to identify genomic lesions in this high-risk subtype of B cell ALL. Studies have shown that NGS is a vital technique to identify various genomic lesions at diagnosis and throughout the treatment that can be missed by the widely used current methods. NGS has improved our understanding of various genomic lesions associated with Ph-like ALL and has helped define disease pathogenesis, MRD evaluation, and stratify therapy to prevent over or under treatment. We are in the era of precision medicine. Therefore unbiased, comprehensive genomic characterization of Ph-like ALL is important to implicate treatment directed against these genomic lesions and improve outcomes in these patients. We also analyzed data from studies that compared NGS with multi-flow cytometry and RQ-PCR for the evaluation of MRD. In the future, more extensive prospective studies are required to confirm the prognostic usefulness of NGS.

## Introduction and background

Acute lymphoblastic leukemia (ALL) accounts for less than 0.5% of all cancers in the United States. In 2019, about 5,930 new cases (3,280 males and 2,650 females) of ALL were diagnosed in the United States. In the same year, there were 1,500 deaths (850 males and 650 females) due to ALL [1]. 

ALL arises from the malignant transformation of B and T lymphoid precursor cells in the bone marrow, and extramedullary sites. ALL is triggered by a variety of genetic mutations, including chromosomal translocation and aneuploidy responsible for cell cycle regulation and lymphoid cell development. ALL is the most common childhood cancer, accounting for 80% of cases, with a five-year survival rate of about 90% in children and 75%-85% in adolescents and young adults [2,3]. It accounts for 15%-25% of all adult leukemias. When occurring in adults, it represents a devastating disease, with an overall five-year survival rate of 35%-55% in middle-aged adults and less than 30% in those over the age of 60 [4]. Despite a 90% cure rate in the pediatric population, it is the critical cause of morbidity and mortality in children and adults [5].

Over the past few decades, there has been an advancement of different technical innovations for the diagnosis of ALL, like quantitative polymerase chain reaction (Q-PCR), pyrosequencing, microarrays, single nucleotide polymorphism (SNP), and digital droplet polymerase chain reaction (dd-PCR). Of these innovations, the most considerable contribution has come from next-generation sequencing (NGS). NGS enables the generation of genomic sequencing information in a relatively shorter length of time with greater precision, that can impact the clinical decision making. It is both sensitive and specific, generates more data with a smaller sample, it is faster, more efficient and its cost is rapidly decreasing. The concept of NGS involves series of massively parallel sequencing through various approaches such as targeted gene sequencing, whole-genome sequencing (WGS), which can reveal structural variations (SVs), whole-exome sequencing (WES) that is useful for detecting point mutations, transcriptome sequencing (RNA-seq) which is used to analyze the expression of mRNA or non-coding RNA and can also identify sequence mutation and fusion genes [6]. 

NGS has led to the identification of many newer molecular entities of ALL and has also provided a more profound understanding of the ones that are already known [7]. Both B cell ALL and T cell ALL are comprised of multiple subtypes defined by structural DNA alterations as an initiating lesion, with secondary somatic (tumor acquired) alterations and sequence mutation, which jointly contribute to leukemogenesis. Structural alterations include aneuploidy and chromosomal rearrangements that can result in the expression of chimeric fusion genes. Sequence mutations commonly alter lymphoid development, cytokine receptors, kinase, and RAS signaling, tumor suppression, and chromatin modification [5].

B cell ALL represents 75% of all cases of ALL and is comprised of various molecular subtypes [2]. In 2016, a new subtype of B-cell ALL was recognized by WHO classification of myeloid neoplasm and acute leukemia; it was called BCR-ABL1-like or Philadelphia chromosome-like (Ph-like) B cell ALL [8]. It was first detected by Mullighan and his colleagues from the Children’s Oncology Group (COG) and St. Jude Children’s Research Hospital (SJCRH), and den Boer and Colleagues from the Netherlands in 2009. Ph-like ALL has a gene expression profile similar to BCR-ABL1 but lacks BCR-ABL1 expression [9]. The Hallmark of Ph-like ALL is the high frequency of IKAROS family zinc finger one (IKZF1) alteration that is 70%-80% as compared to non-Ph-like ALL that is 15%. It is associated with high-risk clinical features, inadequate response to induction therapy, high frequency of persistent minimal residual disease (MRD), and poor outcome, with a five-year disease-free survival of about 60% [10-14].

Transcriptome sequencing studies have shown that Ph-like ALL have a significant genetic heterogeneity with > 70 discrete alterations that dysregulate several classes of cytokine receptors and tyrosine kinases [11,15]. In clinical practice, genetic analysis is conducted through the low throughput techniques like low-density arrays (LDA), fluorescence in situ hybridization (FISH), and Q-PCR. These techniques identify a limited number of detectable alterations but are still primarily used worldwide [7]. NGS has improved our understanding of disease pathogenesis and has also helped identify key biomarkers that are of diagnostic and prognostic importance. Ph-like ALL is a challenging subgroup due to its highly heterogeneous background. Standardized diagnostic tests and clinical management of this subgroup are also not well defined [15,16].

MRD is defined as a state in which leukemic cells remain after treatment. MRD measurement is the single most important factor in patients with ALL that can be used to stratify risk, determine prognosis, and to guide the treatment. Since it has been implemented to stratify treatment strategies, the cure rate has been improved in all age groups. Methods that are widely used in clinical practice to measure MRD are multi-parametric flow cytometry and Q-PCR. Studies have shown that these methods can give false-positive results, and measurement of MRD cannot rely solely on these methods. With the advent of NGS, more attention has been shown to NSG-MRD assay, as it is more sensitive and specific as compared to other methods [17,18].

It is essential to understand the benefits of NGS in clinical practice and to incorporate information gathered through NGS to improve risk stratification and new drug discoveries from the perspective of precision medicine. In this comprehensive review, we will highlight the benefits of NGS in disease definition, risk stratification, and treatment strategies in patients with Ph-like B cell precursor ALL. We will also briefly discuss the studies showing the benefits of NGS in disease definition and MRD monitoring.

## Review

Method

PubMed and PMC online databases were used exclusively for the collection of corresponding data. Results yielded 219 scientific papers based on keywords, and all were in English. After applying specific inclusion-exclusion criteria, 28 scientific papers were deemed relevant. All 28 articles met the quality specification and were peer-reviewed. 

Inclusion and exclusion criteria

All selected scientific papers were written in English and included data collected and reviewed from 2010-2020. The studies that were excluded did not match the inclusion and exclusion criteria. 

Result

Ph-like ALL is a high-risk subtype of B cell ALL. Current methods used to diagnose this high-risk subtype include LDA, Q-PCR, FISH, and flow cytometry. In the last few years, the application of NGS has proved to be an essential diagnostic tool in identifying genomic lesions with higher sensitivity and less bias. Of the four selected research papers, all used NGS as one of the diagnostic tools to identify various genomic lesions in Ph-like ALL. Robert et al. studied Ph-like ALL in children, adolescents, young adults, and older adults. Whereas, Reshmi et al., studied disease in children and young adults and Herold et al. studied Ph-like ALL in adult patients. Studies showed that NGS is useful in identifying the complete repertoire of the leukemic and the normal cells. It can identify new fusions and the splice variants of known lesions during the course of the disease. Despite heterogeneity of Ph-like ALL, most of the treatable genomic lesions can be identified by conventional methods like LDA, Q-PCR, and FISH, however, these methods can only identify known fusions [10,14,19,20]. Table 1, summarizes some of the studies from the review. 

**Table 1:** Details of some of the studies from the review Abbreviations: LDA: low-density array, PCR: polymerase chain reaction, FISH: fluorescence in situ hybridization, SNP: single-nucleotide polymorphism

Author/ Publication year	Country	Study Period	Sample size	Population	Method use	Conclusion
Robert et al. [10], 2014	USA, Canada, Australia New Zealand, Switzerland	1993-2020	6967; 2013 had B cell ALL; 1725 selected; 264 Ph-like ALL	1-65 years; 1-39 years selected	NGS, reverse transcriptase PCR, microarrays, FISH	NGS was performed in 154 patients with Ph-like ALL (30 with CRLF2 and 124 with no CRLF2) and 160 patients with non-Ph-like ALL. The genetic alteration was identified in 91% of patients. CRLF2-20%, JAK2 and EPOR-18%, other JAK-STAT activating sequence mutation-20%, ABL class fusion-22%, RAS pathway mutation-10%, other kinase mutations-1% and no kinase mutation-9%.
Reshmi et al. [19], 2017	USA	2010-2014	1389; 341 had Ph-like ALL, 284 selected	1-30 years	LDA, RT-PCR, FISH, transcriptome sequencing	Kinome capture and RNA seq. were performed in 160 patients with Ph-like ALL (129 with CRLF2 low and 31 with CRLF2 high but lacking CRLF2 rearrangement). Genetic alterations were identified in 83.8% of patients. CRLF2- 43.7%, JAK2 and EPOR- 8.8%, other JAK-STAT seq. Mutation-4.6%, ABL class fusion-14.1%, RAS pathway mutation-6%, other kinases-4.6% and no kinase is ~15%.
Robert et al. [14], 2017	USA, Italy, Canada, Israel, Puerto Rico	1999-2021	3474; 909 had B cell ALL; 798 suitable for genomic analysis; 194 had PH-like ALL	21-86 years	LDA, quantitative reverse transcriptase PCR, FISH, transcriptome sequencing, SNP	Transcriptome sequencing was performed in 86 patients with Ph-like ALL. Genetic alterations were identified in 88% of patients. CRLF2-51%, JAK2/EPOR-12.4%, other JAK-STAT seq. mutation-7.2%, ABL class fusion-9.8%, RAS pathway mutation-3.6%, other kinase-4.1%, and no kinase-4.6%.
Herold et al. [20], 2017	Germany	1999-2008	1475; 207 had B cell ALL, 26 had Ph-like ALL	15-65 years	Immunophenotyping, FISH, amplicon sequencing, Q-PCR	Targeted amplicon sequencing was performed in 39 patients (16 with Ph-like ALL and 23 with B other ALL). RNA and cDNA were not available for further analysis, especially for JAK and ABL fusion.

MRD directed treatment is recommended for Ph-like ALL to avoid over or under treatment due to its heterogeneity and poor outcome. Several studies have been done to compare current methods: multi-parametric flow cytometry (MFC), RQ-PCR based on allele-specific oligonucleotide (ASO), and newly emerging high throughput next-generation sequencing (HT-NGS) for the diagnosis of minimal residual disease. Of the five selected research papers, all confirmed that NGS-MRD detection before transplantation could be useful in improving treatment strategies. After transplantation it allows for early intervention to prevent relapse. Pulsipher et al. in children and Torra et al. in adults compared MFC and HT-NGS. In both the studies, NGS-MRD predicted relapse and survival more accurately than MFC-MRD. Patients without pre-hematopoietic stem cell transplant (pre-HSCT) NGS-MRD had a very low risk of relapse with an event-free survival of 96%, which indicates the possibility of altering therapeutic approaches in these patients. Whereas positive NGS-MRD in post HSCT patients had a higher risk of relapse [21,22]. Kotrova et al. and Ladetto et al. compared NGS-MRD with RQ-PCR and demonstrated good concordance between two and higher sensitivity with NGS as compared to RQ-PCR without the need to identify patient-specific probes [23,24]. Faham et al. compared all three methods and confirmed that NGS could detect residual disease at levels below one in one million leukocytes (0.0001%), which represents one-two folds of the higher sensitivity of NGS than the gold standard methods [25]. Table 2 summarizes some of the MRD studies from the review.

**Table 2:** Details of some of the MRD studies from the review Abbreviations: NGS, next-generation sequencing; RQ-PCR, real-time quantitative polymerase chain reaction; MFC, multi-parametric flow cytometry; MRD, minimal residual disease; ASO, allele-specific oligonucleotide; OS, overall survival

Author/ publication year	Country	Study period	Sample size	Population	Method use	Conclusion
Faham et al. [25], 2012	USA	2000-2014	498; 110 selected	1-18 years	NGS, MFC, RQ-PCR	NGS measured treatment response with greater sensitivity and precision as compared to MFC and ASO-PCR. NGS and MFC: Concordance is seen in 90%. NGS and PCR: concordance is seen in 96%
Ladetto et al. [24], 2014	Europe	2003-2018	2308; 55 selected	15 and older	NGS and RQ-PCR	Concordance in 79.6%, indicating that NGS is more sensitive as compared to RQ-PCR and does not require a patient-specific probe.
Pulsipher et al. [21], 2015	USA, Canada, Australia	2007-2011	146; 56 selected	1-21 years	NGS and MFC	Negative Pre-HCT: NGS-MRD-2 yr. Relapse probability 0% and OS 96%. MFC-MRD- relapse 16% and OS 77%. Positive Pre-HCT: NGS-MRD-2 yr. Relapse probability 53% and OS 48%, MFC-MRD- relapse 46%. Post-HCT (30 days): NGS-MRD positive-relapse 67%, MFC-MRD positive-35% NGS-MRD negative-relapse 25%
Torra et al. [22], 2017	USA	2005-2014	78; 32 selected	18-64 years	NGS and MFC	Relapse free survival (RFS): NGS-MRD negative (P=0.018) as compared to MFC-MRD negative (P=0.0032). Concordance in 82% and discordance in 17%, indicating that NGS is more sensitive and accurate than MFC
Kotrova et al. [23], 2017	Europe	2000-2014	30	1-18 years	NGS and RQ-PCR	NGS was found to be highly specific in post stem cell transplant management as compared to RQ-PCR

Discussion

Ph-like ALL

Ph-like ALL is a high-risk subtype and accounts for 20% of all B cell ALL cases. Its prevalence differs by age, gender, ethnicity and National Cancer Institute (NCI)-defined risk groups. It comprises of approximately 10% in children of NCI standard risk (age:1-9 years and white blood cell (WBC) count <50 x 10^9^/L), 13% in children of NCI high risk ( age: 10-15 and WBC count >50 x 10^9^/L), 21% of adolescents (16-20 years), 27% of young adults (21-39 years) and 20%-24% of adults over 40 years of age. It is more common in males with male to female ratio is 2:1 as compared to 1.6:1 in non-Ph-like ALL. It is highly prevalent in Hispanic patients due to presence of germline variant GATA3 in Hispanic and native American genetic ancestry, particularly common with Ph-like ALL with cytokine receptor like factor two-rearrangement (CRLF2-R). Ph-like ALL is a frequently occurring subtype in pediatric and young adults, and is associated with poor response to induction chemotherapy, elevated WBC count, high levels of MRD. Overall survival (OS) and event free survival (EFS) across all age group is inferior as compared to non-Ph-like ALL. It is important to identify these high-risk patients who could potentially benefit from targeted therapy [10,14,19]. 

Ph-like ALL is similar to BCR-ABL1 ALL in gene expression profile but lacks BCR-ABL1 fusion protein expressed from the t(9;22) (q34; q11.2). Ph-like ALL with IKZF1 alteration has an inferior five-year EFS as compared to Ph-like ALL with no IKZF1 alteration. IKZF1 alterations are more common in patients with kinase mutation as compared to patients with sequence mutation [9-14]. Approximately half of the Ph-like ALL cases have CRLF2-R with concomitant Janus kinase one/Janus kinase two (JAK1/JAK2) mutation. Recent introduction of transcriptome sequencing and whole genome sequencing in patients without CRLF2-R, have identified kinase and cytokine receptor signaling alteration in Ph-like ALL patients. These include, erythropoietin receptor (EPOR), ABL-class fusion, RAS pathway mutations, uncommon kinase mutation and sequence mutations activating JAK-STAT pathway [26]. 

Identification of Ph-like ALL

Clinical and research laboratories around the world uses different methods to identify Ph-like ALL. In Europe, multiplex PCR or targeted RNA sequencing or FISH is used by different groups. In the United States, clinical oncology group (COG) uses LDA for screening, whereas only some centers in the USA uses comprehensive RNA sequencing, like St. Jude Medical Center [27]. Several studies have used NGS on the available samples to identify genetic alteration activating kinase or cytokine receptor signaling. Number of these genomic lesions can be treated with the FDA approved tyrosine kinase inhibitors (TKIs).

Roberts et al. studied patients ages 1-39 years from multi-center clinical trials conducted between 1993 and 2020, and it included 6967 total participants [10]. They selected 2,013 patients who had precursor B cell ALL, 1,725 of whom had material available for microarray gene-expression profiling. A total of 264 of 1,725 (15.3%) were identified as having Ph-like ALL. CRLF2 expression was identified in 123 of 264 patients (24% of children with NCI standard risk and 60% of adolescence). A total of 154 patients (30 with CRLF2 and 124 patients without CRLF2 rearrangement) with Ph-like ALL underwent one or more different types of NGS, like transcriptome sequencing, whole genome sequencing, and whole-exome sequencing. NGS was also performed in 160 patients with non-Ph-like ALL. Genomic analysis identified kinase signaling alterations in 91% of patients with Ph-like ALL, and they were divided into distinct subgroups. These included JAK-STAT mutations, ABL class fusion, RAS pathway mutations, other uncommon mutations, and no kinase alterations [10]. These are discussed later in this section. 

Reshmi et al. also studied patients in the age group 1-30 years [19]. They retrospectively identified 1389 patients with B cell ALL from clinical trials between 2010 and 2014. All patients were analyzed by using a low-density array (LDA). LDA identified 341 patients with Ph-like ALL, 1048 LDA negative were excluded. Application of LDA in pediatric patients failed to identify mutations in 15% of LDA positive cases but identified nine patients with CRLF2 rearrangement who were LDA negative. Out of 341, 57 patients with BCR-ABL positive and ETV6-RUNX1 were excluded for further analysis. A total of 284 patients were included for the final analysis. Genetic alteration activating kinases and cytokine receptor signaling was identified in 238 of 284 patients (83.8%). CRLF2 rearrangement was identified in 124 out of 284 patients. The remaining 160 patients with no CRLF2 rearrangement underwent reverse transcriptase PCR initially to identify kinase alterations in known fusion and, if negative underwent Kinome capture and RNA sequencing. It identified various alterations that are discussed later in this section. 

Roberts et al. also studied Ph-like ALL in adult patients aged 21-86 years [14]. They retrospectively identified patients from multi-center clinical trial between 1999 and 2021. These trials included 3474 total participants, 909 patients were diagnosed with B cell ALL, 798 of whom were selected for this study due to the availability of suitable material for genomic analysis. Ph-like ALL was identified in 194 out of 798 patients. Out of 194, 180 patients were selected for genomic analysis. CRLF2 expression was identified in 99 out of 194 patients. Eight six of the remaining 95 patients underwent transcriptome sequencing. Kinase and cytokine receptor signaling alterations were identified in 88% of patients with Ph-like ALL. They were divided into subtypes that are discussed later in this section. Eleven new genetic alterations of B ALL were also identified during this study. 

Herold et al. also studied Ph-like ALL in adult patient aged 15-65 years [20]. They identified 207 patients with B cell ALL from two clinical trial between 1999 and 2008. A total of 1475 patients were included in these trials. Ph-like ALL was diagnosed in 26 out of 207 patients using gene expression profiling. Targeted amplicon sequencing was performed on 131 genes that were recurrently mutated in 16 patients with Ph-like ALL and 23 patients with remaining BCP-ALL. 

All studies concluded that Ph-like ALL has a large number of complex genomic lesions that can activate a limited number of signaling pathways, and many of these are amenable to tyrosine kinase inhibitors. Despite of complex nature of Ph-like ALL, majority of treatable genomic lesions can be rapidly and accurately identified by the conventional methods like LDA, FISH and Q-PCR. These methods are simple and cost effective, but can only identify known fusions. Newer fusions or splice variants of known fusions cannot be identified with conventional methods; therefore, patients with B cell ALL at screening or those identified as having Ph-like ALL should undergo unbiased RNA sequencing. It can identify all genomic lesions in a comprehensive and timely manner. It is not feasible to incorporate NGS for diagnosis of Ph-like ALL, particularly in the clinical trials involving large number of patients from multiple sites, but its declining cost and improvement in bioinformatic analysis in future can make NGS a powerful tool to identify genomic lesions and help in precision medicine. 

Kinase Alterations in Ph-like ALL

Gene expression profiling has identified kinase and cytokine receptor signaling alteration in Ph-like ALL. They are divided into four subtypes: JAK-STAT mutations, ABl class fusion, RAS pathway mutation, and uncommon kinase mutation [10,14]. 

JAK-STAT signaling: It is the largest class of kinase activation lesion that can result in the activation of Janus kinase signal transducer and activator of transcription (JAK-STAT) signaling. Half of the mutation is a rearrangement of cytokine receptor-like factor two (CRLF2). CRLF2 is dysregulated by translocation of the immunoglobulin heavy chain (IGH-CRLF2) or focal deletion resulting in the formation of P2RY8-CRLF2. Both of these can result in the expression of CRLF2, which heterodimerizes with interleukin seven receptor alpha (IL7RA) to form the thymic stromal-derived lymphopoietic receptor (TSLPR) [28]. Less commonly, there is a sequence mutation of CRLF2 (F232C). CRLF2-R is common in Ph-like ALL and patients with down syndrome, and it is age-dependent with IGH-CRLF2 common in older age and Hispanic ancestry, whereas P2RY8 is seen in young age. Most studies have shown that CRLF2 is associated with poor prognosis primarily when related to concomitant IKZF1 alteration. The majority of CRLF2 have concomitant alteration, particularly JAK1 or JAK2, and are associated with the poorest outcome. Other genetic alterations that activate JAK-STAT signaling are Janus kinase two (JAK2) and erythropoietin receptor (EPOR). There are sequence mutations and DNA copy number alterations that activate the JAK-STAT signaling pathway but have no kinase or cytokine receptor gene rearrangement. These include Janus kinase one (JAK1), JAK2, interleukin seven receptor (IL7R), Src homology two adaptor protein three (SH2B3), interleukin two receptor beta (IL2RB), and tyrosine kinase two (TYK2). All these above-mentioned genetic lesions activate the JAK-STAT signaling pathway and can be treated with JAK inhibitors like ruxolitinib [9,10,14,28]. 

ABL class fusion: Kinases that are altered in this subtype of Ph-like ALL are Abelson murine leukemia one (ABL1), ABL2, colony-stimulating factor one receptor (CSF1R), platelet-derived growth factor receptor A (PDGFRA), and PDGFRB. These kinases can be inhibited by ABL1 inhibitors like imatinib and dasatinib [10,14]. 

RAS pathway: Ph-like ALL have a mutation in rat sarcoma viral oncogene/mitogen-activated protein kinase (Ras/MAPK) signaling pathway. It does not inhibit Ras directly, but it inhibits a molecule called methyl-ethyl ketone (MEK). Genomic lesions that can activate the RAS pathway include Kristen rat sarcoma viral oncogene (KRAS), neuroblastoma rat sarcoma viral oncogene (NRAS), neurofibromatosis type one (NF1), protein tyrosine phosphatase non-receptor type 11 (PTPN11), and cerebellin one (CBL1). This class can be targeted with FDA-approved tramatinib, selumetinib, cobimetinib [10,14]. 

Less common kinase alterations: This subtype of Ph-like ALL include neurotrophic tyrosine receptor kinase three (NTRK3), B cell linker (BLNK), protein tyrosine kinase two beta (PTK2B) and fms-like tyrosine kinase three (FLT3). NTRK3 targetable with crizotinib, PTK2B with focal adhesion kinase (FAK) inhibitors and TYK2 with TYK2 inhibitors [10]. Table 3 summarizes subtypes of Ph-like ALL. 

**Table 3:** Subtypes and therapeutic targets of Ph-like ALL Abbreviations: JAK-STAT, Janus kinase signal transducer and activator of transcription; Ph-like ALL, Philadelphia chromosome-like acute lymphoblastic leukemia

Subtype	Alteration	Treatment	Comments
JAK-STAT signaling [9,10,14,28]	CRLF2 (translocation IgH-CRLF2, focal deletion P2RY8-CRLF2 and sequence mutation F232C) , JAK2, EPOR and sequence mutation (JAK1, JAK2, IL7R, SH2B3, IL2RB, TYK2)	Ruxolitinib	CRLF2 50%, common in Ph-like ALL and down syndrome, poor prognosis, IGH-CRLF2-older age and Hispanic and P2RY8-CRLF2-young age
ABL class fusion [10,14]	ABL1, ABL2, CSF1R, PDGFRA, PDGFRB	Imatinib, dasatinib	
RAS pathway [10,14]	RAS/MAPK, KRAS, NRAS, NF1, PTPN11, CBL1	Tramatinib, selumetinib, Cobimetinib	
Less common alterations [10]	NTRK3, BLNK, PTK2B, FLT3	NTRK3-Crizotinib, PTK2B-FAK inhibitors, FLT3-FLT3 inhibitors and TYK2-TYK2 inhibitors	

**Figure 1:**
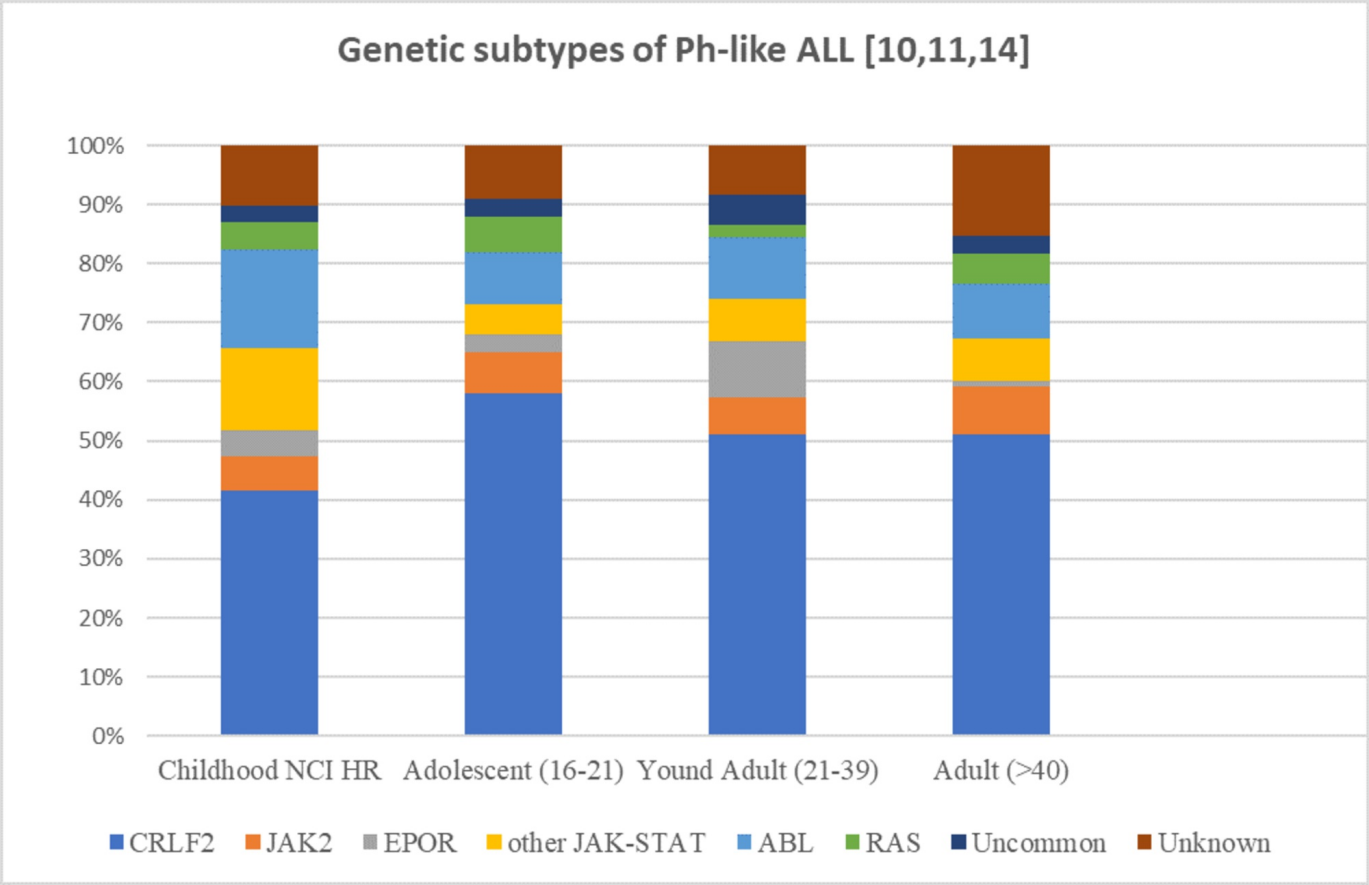
Frequency of genetic subtypes in patients with Ph-like ALL by age group Abbreviations: NCI: National Cancer Institute, HR: high risk (age:10-15 and WBC count >50 x 10^9^/L); Ph-like ALL: Philadelphia chromosome-like acute lymphoblastic leukemia

Figure 1 shows the frequency of genetic subtypes in patients with Ph-like ALL by age group.

Measurement of MRD

MRD measurement is performed to evaluate treatment response and to define risk stratification. Undetectable MRD after induction therapy indicate good prognosis, whereas persistent MRD indicates a high risk of relapse. MRD analysis should be accurate, sensitive (≤0.01% or ≤10^-4^) and specific. MRD is diagnosed by targeting molecular rearrangements like gene fusion, immunoglobulins (IgH), and T cell receptor (TCR) gene. Current methods used to measure MRD are multi-parametric flow cytometer (MFC), ASO-PCR, and NGS of the IgH VDJ and/or TCR gene rearrangement. Ph-like ALL is associated with persistent MRD levels and poor response to therapy [21-25]. It is crucial to accurately measure MRD in these high-risk subtype patients to identify MRD levels and treat accordingly. Several studies have been done to compare NGS, MFC, and RQ-PCR for the measurement of residual disease.

Pulsipher et al. compared NGS-MRD assay and MFC-MRD assay to predict relapse and overall survival (OS) in pre-HCT and post-HCT patients. They identified a cohort of uniformly treated patients from multi-center clinical trials by the children oncology group (COG) conducted between 2007 and 2011. They included patients ages 1-21 years. A total 146 participants were included in these trials. Pre-hematopoietic cell transplant (pre-HCT) samples (blast and bone marrow) were available for 79 out of 146 subjects. B-cell ALL was identified in 66 out of 79 patients and 56 out of 66 subjects had detectable immunoglobulin heavy chain (IgH) variable (V), diversity (D) and joining (J) tagging sequence. In 15 out of 56 pre-HCT BM sample was not available. A total of 41 subjects were included for final analysis for pre-HCT NGS-MRD assay and 40 out of 41 also underwent pre-HCT MFC-MRD assay. Out of 56 with detectable IgH VDJ tagging sequence, 53 were selected for post-HCT NGS-MRD assay and 52 out of 53 also underwent MFC-MRD assay. Study showed that NGS is superior to MFC for the prediction of relapse (P<.0001) in both pre and post hematopoietic cell transplant patients and was better at predicting overall survival (OS) (P=.003) [21]. 

Torra et al. identified adult patients of ages 18-64 years from a single multi-center trial conducted between 2005 and 2014. A total of 78 subjects were included in this trial. Patients included in this study were 32 whom pre-treatment and follow-up bone marrow (BM) and peripheral blood (PB) samples were available. A total of 29 subjects were identified for the final analysis as three subjects were excluded due to lack of detectable pre-treatment IgH VDJ sequence. Data for NGS-MRD and MFC-MRD both was collected from 25 patients (66 samples). In pre-treatment samples, there was strong correlation between two methods, whereas post-treatment MRD results showed discordances (33% samples positive by NGS were negative by MFC, but no samples positive by MFC were negative by NGS) [22]. 

Kotrova et al. compared NGS and Q-PCR for the detection of residual disease. They identified 30 patients ages 1-18 years from a clinical trial conducted between 2000 and 2014. Patients were selected based on DNA sample availability, MRD positive by Q-PCR and patient who remained in complete remission for at least two years. B cell ALL was identified in 28 patients and two had T-cell ALL. Q-PCR was conducted on 228 BM and PB samples, 42 samples out of 228 also underwent NGS. NGS is specific in identifying residual disease post-HCT as Q-PCR can give false-positive results, therefore, treatment strategies cannot be based on Q-PCR alone [23]. 

Ladetto et al. also compared NGS with Q-PCR to detect MRD in patients with ALL, multiple myeloma (MM) and mantle cell lymphoma (MCL). Patients ages 15 and older were identified from clinical trials between 2003 and 2018. A total of 2,308 patients participated in these trials. Out of 2308, 55 patients were selected for this study (15 ALL, 30 MCL, 10 MM). Total of 378 samples were collected (218 BM and 160 PB), 62 at diagnosis and 316 after treatment. In 15 ALL patients, 26 samples underwent both NGS-MRD and Q-PCR-MRD assay. Both tools reached a sensitivity of 10^-5^, showed good correlation (R=0.791, P<0.001), concordance of 79.6% and 15% discordant cases [24]. 

Faham et al. compared NGS-MRD with MFC-MRD and ASO-PCR in patients ages 1-18 years. They identified patients from clinical trial conducted between 2000 and 2014. A total of 498 patients participated in this trial. Out of 498, 110 children had B cell ALL. BM samples from 110 children were collected at diagnosis and during treatment. Samples of 106 children underwent NGS, MFC and Q-PCR for the measurement of residual disease. NGS can detect all genomic rearrangements at diagnosis and during the course of disease and treatment as compared to Q-PCR, this can reduce the risk of false-negative MRD results, that occur due to clonal evolution during the course of disease and treatment [25]. 

Above studies, concluded that, undetectable pre-HCT IgH VDJ NGS-MRD have a very low risk of relapse, whereas detectable post-HCT IgH VDJ NGS-MRD have a high risk of relapse. NGS-MRD is superior to MFC-MRD in predicting overall survival, relapse and no relapse in pre-HCT and post-HCT patients. If the patient has no pre-HCT NGS-MRD, they are eligible for less intense treatment strategy. NGS is more sensitive than MFC in the detection of residual disease and can identify patients that might benefit from transplant. Due to increased sensitivity of NGS, peripheral blood samples can complement bone marrow samples, for the detection of residual disease, as data from Torra et al. showed that only 17% of cases were not detected by NGS PB samples, but these cases had low MRD levels in bone marrow. NGS is more specific than Q-PCR in predicting relapse in post-transplant patients, as data indicates that Q-PCR can give false-positive results that can lead to over treatment. NGS sensitivity is comparable with Q-PCR but without the need for patient-specific probes. Studies showed pre-treatment high concordance between these methods. There were some discordances post-treatment due to clonal evolution that are not detected by MFC or Q-PCR at an earlier time point as compared to NGS. Table 4 summarizes the advantages and disadvantages of NGS, RQ-PCR and MFC. 

**Table 4:** Advantages and disadvantages of NGS, MFC, Q-PCR Abbreviations: NGS, next-generation sequencing; MFC, multi-parametric flow cytometry; Q-PCR, quantitative polymerase chain reaction

	MFC	RQ-PCR	NGS
Advantages [21-25]	Easy, fast, cost-effective, Widely used	Has sensitivity of ~10^-4^-10^-5^, Widely used	Higher sensitivity >10^-6^, due to increase sensitivity MRD can be detected in PB avoiding painful BM samples, it does not require patient specific probes, it identifies all genomic rearrangements at diagnosis and their evolution during treatment and it can detect MRD at an earlier time (30 day) after HCT
Disadvantages [21-25]	It does not always reach sensitivity of 10^-4^ (1 ALL blast in 10^-4^ normal cells), lack of standardization across testing site, different inter-sample sensitivity, cannot detect evolution of new fusion or variants of known fusion during treatment, it requires expertise, poor ability to predict relapse early after HCT and lack of specificity	Time consuming, costly, laborious, require different primer and protocol for different patients, cannot detect evolution of new fusion or variant of known fusion during treatment and lack of specificity.	Expensive, require expertise, lack of standardization, require highly efficient bioinformatics and large number of data is generated to be stored and analyzed.

Limitations

Our study had faced a few limitations as described below:

1. Most of the clinical studies included pediatric, adolescence and young adults with some studies focusing on older adults.

2. Despite having found a large number of sample sizes, it would be beneficial if NGS have been conducted on a larger percentage of the chosen sample size patients.

3. Every study utilized a different approach to NGS. 

4. We selected studies conducted in the English language only that might have caused us to miss some more valuable reviews.

## Conclusions

This review article aimed to analyze and assess the importance of NGS in genomic characterization of PH-like ALL and MRD monitoring. It was shown: (1) Ph-like ALL has a low survival in all age groups and is highly heterogenous subtype of B cell ALL. More accurate method like NGS is required for the genomic characterization of Ph-like ALL early in the disease, to improve outcomes in these patients. Analysis of these fusions should not rely only on conventional methods, as they can miss some and/or many targetable alterations. (2) Ph-like ALL is associated with persistent MRD levels and high risk of relapse. Clinical studies have shown that NGS is highly accurate, sensitive, specific and can measure MRD levels early after the induction therapy that can be missed by other methods used. (3) Implementation of NGS in the clinical trials have broadened our understanding of disease pathogenesis, allowing careful patient management in regard to prognosis, treatment choice, MRD evaluation and new drug discoveries. 

Our findings support that in the future, all patients with B cell ALL should be considered to undergo expression-based screening at diagnosis either with LDA, RQ-PCR or FISH to identify phenotype, followed by the search of targetable genotype and measurement of MRD levels during the treatment with any of the various approaches of NGS. We believe that in the future, we will see larger prospective studies implementing NGS across all age groups, that will completely describe the genomic landscape and will help refine targeted and individualized therapy in this high-risk subtype of B cell ALL.
